# Impact of race on outcomes from catheter ablation of ventricular tachycardia in structural heart disease: A prospective registry from south metropolitan Chicago

**DOI:** 10.1016/j.hroo.2023.01.007

**Published:** 2023-01-31

**Authors:** Nathan W. Kong, Dalise Y. Shatz, Stephanie A. Besser, Gaurav A. Upadhyay, Roderick Tung

**Affiliations:** ∗ Department of Internal Medicine, University of Chicago, Chicago, Illinois; † Center for Arrhythmia Care, Pritzker School of Medicine, University of Chicago, Chicago, Illinois

**Keywords:** Catheter ablation, Health care disparities, Prospective registry, Race, Ventricular tachycardia

## Abstract

**Background:**

Whether racial disparities in outcomes are present after catheter ablation for scar-related ventricular tachycardia (VT) is not known.

**Objective:**

The purpose of this study was to examine whether racial differences exist in outcomes for patients undergoing VT ablation.

**Methods:**

From March 2016 through April 2021, consecutive patients undergoing catheter ablation for scar-related VT at the University of Chicago were prospectively enrolled. The primary outcome was VT recurrence, with secondary outcome of mortality alone and composite endpoint of left ventricular assist device placement, heart transplant, or mortality.

**Results:**

A total of 258 patients were analyzed: 58 (22%) self-identified as Black, and 113 (44%) had ischemic cardiomyopathy. Black patients had significantly higher rates of hypertension (HTN), chronic kidney disease (CKD), and VT storm at presentation. At 7 months, Black patients experienced higher rates of VT recurrence (*P* = .009). However, after multivariable adjustment, there were no observed differences in VT recurrence (adjusted hazard ratio [aHR] 1.65; 95% confidence interval [CI] 0.91–2.97; *P* = .10), all-cause mortality (aHR 0.49; 95% CI 0.21–1.17; *P* = .11), or composite events (aHR 0.76; 95% CI 0.37–1.54; *P* = .44) between Black and non-Black patients.

**Conclusion:**

In this diverse prospective registry of patients undergoing catheter ablation for scar-related VT, Black patients experienced higher rates of VT recurrence compared to non-Black patients. When adjusted for highly prevalent HTN, CKD, and VT storm, Black patients had comparable outcomes as non-Black patients.

**Key Findings**

▪ Black patients undergoing ventricular tachycardia (VT) ablation had higher unadjusted risk of VT recurrence at follow-up.
▪ Black patients undergoing VT ablation had higher rates of hypertension and chronic kidney disease, and were more likely to present in VT storm.
▪ After adjustment for comorbidities including hypertension, chronic kidney disease, and VT storm, there was no difference in VT recurrence between racial groups.

## Introduction

Catheter ablation is the treatment of choice for symptomatic, sustained monomorphic ventricular tachycardia (VT).^1^ Randomized clinical trials and prospective cohort studies have demonstrated efficacy with catheter ablation for VT in the setting of structural heart disease.^2^, ^3^, ^4^ Previous studies have highlighted important differences between men and women undergoing VT ablation, and racial differences have been described in outcomes after catheter ablation for other arrhythmias such as atrial fibrillation.^5^^,^^6^ Previous studies of VT ablation have not included a sufficient number of racial minorities, particularly patients who self-identify as Black, to examine whether any meaningful differences in outcomes exist between races. We analyzed a single-center prospective registry from south metropolitan Chicago to determine whether racial disparities exist between patients who identify as Black and other groups undergoing catheter ablation for scar-related VT.

## Methods

### Patient selection

Consecutive patients between March 2016 and April 2021 with structural heart disease and evidence of electroanatomic scar, defined as low voltage <1.5 mV, were included for the present analysis. Patients with ischemic cardiomyopathy (ICM) and nonischemic cardiomyopathy (NICM) (including arrhythmogenic right ventricular cardiomyopathy) were included. All patients were prospectively enrolled in the University of Chicago VT Ablation Registry (IRB16-0272), which was established to examine the safety and outcomes during and after catheter ablation. If patients underwent multiple ablations, outcomes after the most recent procedure were reported. The University of Chicago Medical Center Institutional Review Board approved the creation, maintenance, and review of this prospective registry. All subjects provided informed consent. The research reported adhered to the Declaration of Helsinki as revised in 2013. Patients without any follow-up were excluded from analysis.

### Catheter ablation

Noninvasive stimulation was performed with patients under light conscious sedation before induction of anesthesia to assess the morphology of a clinical or targeted VT and hemodynamic tolerance. General anesthesia with intubation was administered in all cases. Epicardial mapping and ablation was performed at the discretion of the operator, typically for cases with a history of previously failed endocardial ablation or cardiac magnetic resonance imaging that suggested epicardial delayed enhancement. Intravenous dopamine infusions (5–10 μg/min) were initiated if hypotension occurred or in cases with systolic blood pressure <110 mm Hg before programmed stimulation. High-density electroanatomic maps were created in sinus rhythm or paced rhythm with multielectrode catheters using CARTO (PentaRay, Biosense Webster, Diamond Bar, CA), EnSite Precision (2-2-2 Livewire, Abbott, Abbott Park, IL), or Rhythmia (Orion, Boston Scientific, Natick, MA) with standard low-voltage bipolar settings (0.5–1.5 mV; 0.5–1.0 mV for Rhythmia).

Radiofrequency ablation was performed using an open-irrigated catheter (ThermoCool or ThermoCool SF, 3.5 mm, Biosense-Webster; FlexAbility SE, Abbott; IntelliNav, Boston Scientific). As previously described, high-density mapping with multielectrode catheters was performed to identify wavefront discontinuities in regions with isochronal crowding. Isochronal late activation mapping was performed to annotate local electrograms at offset of latest component. Ablation was performed until local electrograms were reduced or eliminated within deceleration zones with complete noninducibility as the procedural endpoint.^7^

### Clinical data and follow-up

Sex was defined as the one assigned at birth (either male or female). Race was self-identified at the time of registration. Height and weight were determined at the time of VT ablation. Body mass index (BMI) was calculated as the weight (in kilograms) divided by the height (in meters) squared. Obesity was defined as BMI ≥30 kg/m^2^. Left ventricular ejection fraction (LVEF) was determined from the most recent transthoracic echocardiogram before the VT ablation date. VT storm was defined as ≥3 episodes of an episode of VT requiring termination with cardioversion or antitachycardia pacing at the time of presentation. The etiology of the patient’s cardiomyopathy was determined by the treating physician based on clinical history.

Patients were followed routinely with clinical history, physical examination, and implantable cardioverter-defibrillator interrogation within the first month after ablation and every 3–6 months thereafter. VT recurrence was defined as documented sustained monomorphic VT >30 seconds in duration or any appropriate implantable cardioverter-defibrillator therapy with antitachycardia pacing or delivery of shock. Left ventricular assist device (LVAD) implantation, heart transplant, and all-cause death were confirmed with electronic health records, the referring physician, or family members. Patients were followed until VT recurrence, LVAD implantation, heart transplant, death, or most recent clinic visit, as time to first event analysis. Prespecified subgroup analysis by ICM, NICM, and LVEF <40% also was performed. The primary outcome of interest was VT recurrence. The secondary outcome was composite and individual occurrence of LVAD implantation, heart transplant, and all-cause death.

### Statistical analysis

For baseline and clinical follow-up outcomes, categorical variables are given as count (percentage), and continuous variables are given as either mean ± SD (if normally distributed) or median [interquartile ranges] (if non-normally distributed). Variables were compared using the χ^2^ test of association or Fisher exact test for categorical variables and Student *t* test for continuous variables with normal distributions. Continuous variables with non-normal distributions were compared using the Mann-Whitney *U* test. Cox proportion hazard models were created for primary and secondary outcomes. The proportion hazards assumption was checked using the Schoenfeld residual method. Results were adjusted for age, sex, history of hypertension, history of chronic kidney disease (CKD), procedural duration, and presentation of VT storm. Kaplan-Meier survival analysis was performed on primary and secondary outcomes as well as prespecified subgroup analysis for the year following catheter ablation. All 2-tailed *P* <.05 were considered significant. All analyses were performed using Stata Version 17 (StataCorp., College Station, TX), and visualizations were performed with ‘tidyverse’ and ‘survival’ packages in R 4.1.2 (R Core Team, 2021).

## Results

### Baseline characteristics

Between March 2016 and April 2021, 289 ablation procedures for scar-related VT were performed at the University of Chicago Medical Center. After exclusion of repeat procedures, 258 patients (89.3% of total procedures) remained for final analysis. Median age was 65 [58–71] years, and 39 patients (15%) were female. One hundred eighty-nine patients (73%) self-identified as non-Hispanic White, 58 (22%) self-identified as Black, 6 (2%) self-identified as Hispanic White, and 5 (2%) self-identified as Asian/Pacific Islander. Median LVEF was 31% [25%–42%]. One hundred thirteen patients (44%) had ICM as the etiology of their cardiomyopathy. One hundred twenty-two patients (47%) previously had undergone a VT ablation procedure, and 37 (14%) had undergone ≥2 previous ablation attempts. One hundred t patients (43%) presented as VT storm. Median follow-up time was 6.5 [1–19] months.

Stratified by self-identified race, Black patients were more likely to have hypertension (66% vs 40%; *P* = .001), CKD (55% vs 25%; *P* <.001), and present as VT storm (57% vs 40%; *P* = .028) (Table 1). There were no significant differences with regard to age, sex, BMI, baseline LVEF, etiology of cardiomyopathy, previous antiarrhythmic medications, number of previous VT ablations, or total procedural time.

**Table 1:** Baseline characteristics by self-identified race

	Black	Non-Black	*P* value
No. of patients	58	200	
Age (y)	62.5 [53.5–69.0]	65.0 [59.0–71.0]	.128
Male	46 (79.3)	173 (86.5)	.255
Body mass index (kg/m^2^)	28.7 [25.4–32.4]	28.4 [25.2–32.2]	.696
Obesity (BMI >30 kg/m^2^)	23 (43.4)	74 (38.7)	.650
Left ventricular ejection fraction (%)	30.5 [24.1–40.1]	31.9 [25.0–44.2]	.368
Ischemic cardiomyopathy	26 (44.8)	87 (43.5)	.977
Nonischemic cardiomyopathy	32 (55.2)	113 (56.5)	.977
Arrhythmogenic right ventricular cardiomyopathy	2 (3.4)	16 (8.0)	.365
Hypertension	38 (65.5)	79 (39.5)	**.001**
Diabetes mellitus	14 (24.1)	43 (21.5)	.805
Coronary artery disease	32 (55.2)	86 (43.0)	.137
Chronic kidney disease	32 (55.2)	49 (24.5)	**<.001**
Stroke or transient ischemic attack	5 (8.6)	11 (5.5)	.366
Atrial fibrillation or atrial flutter	18 (31.0)	72 (36.0)	.588
VT storm	33 (56.9)	79 (39.5)	**.028**
No. of previous VT ablations			.831
0	34 (58.6)	102 (51.0)	
1	17 (29.3)	68 (34.0)	
2	4 (6.9)	17 (8.5)	
≥3	3 (5.2)	13 (6.5)	
Previous β-blocker	42 (72.4)	121 (60.5)	.133
Previous antiarrhythmic medication	33 (56.9)	114 (57.0)	1
Previous amiodarone	25 (43.1)	75 (37.5)	.536
Implantable cardioverter-defibrillator			.224
Biventricular	10 (17.2)	54 (27.0)	
Dual-chamber	30 (51.7)	80 (40.0)	
Single-chamber	12 (20.7)	44 (22.0)	
Subcutaneous	0 (0.0)	7 (3.5)	
None	6 (10.3)	15 (7.5)	
Mapping access			.556
Endocardial only	31 (53.4)	92 (46.0)	
Epicardial only	2 (3.4)	11 (5.5)	
Endocardial and epicardial	25 (43.1)	97 (48.5)	
Procedural time (min)	300 [255–382]	289.0 [225–362]	.140
Fluoroscopy time (min)	25.0 [14.5–37.2]	18.6 [10.6–30.6]	.123
Ablation time (s)	1672 [1081–2352]	1423 [1024–2101]	.337
I-VT score–VT recurrence risk			.383
High risk	13 (22.4)	35 (17.5)	
Intermediate risk	29 (50.0)	91 (45.5)	
Low risk	16 (27.6)	74 (37.0)	
I-VT score–Mortality risk			.184
High risk	18 (31)	43 (21.5)	
Low risk	40 (69.0)	157 (78.5)	

Categorical variables are given as count (percent). Normally distributed continuous variables are given as mean. Non-normally distributed continuous variables are given as median [interquartile range].

Bold values indicate statistically significant.

BMI = body mass index; I-VT = International Ventricular Tachycardia; VT = ventricular tachycardia.

### VT recurrence

**Figure 1:**
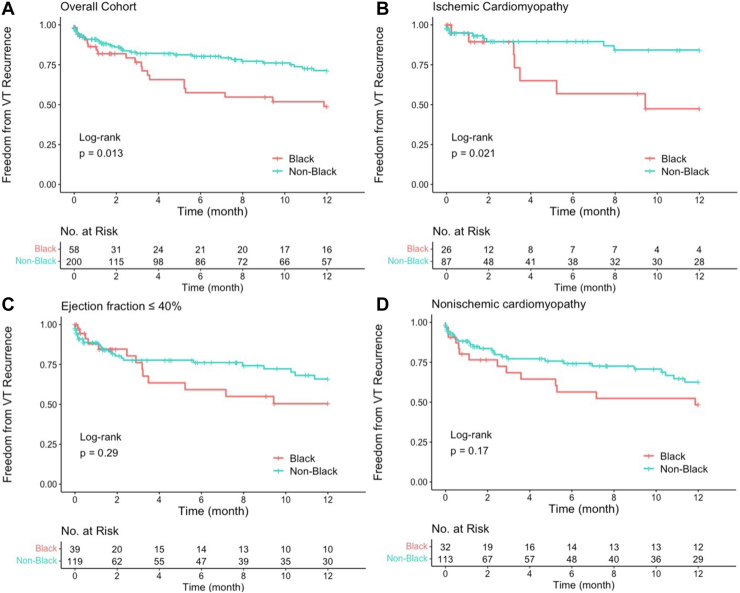
Freedom from ventricular tachycardia (VT) recurrence by self-identified race **(A)** and subgroup analysis of ejection fraction ≤40% **(B),** ischemic cardiomyopathy **(C),** and nonischemic cardiomyopathy **(D).**

In total, 66 patients (26%) experienced VT recurrences. Black patients were significantly more likely to have VT recurrence compared to other races (40% vs 22%; *P* = .009). In unadjusted time-to-event analysis, Black patients had significantly a higher rate of VT recurrence (*P* = .013) in the year after ablation (Figure 1A). Kaplan-Meier subgroup analysis showed no significant difference in VT recurrence in patients with LVEF ≤40% (Figure 1B) and those with NICM (Figure 1D). In those with ICM, Black patients experienced a lower rate of VT-free survival compared to other races (*P* = .021) (Figure 1C). Black patients were at higher risk for VT recurrence compared to other races in unadjusted hazard analysis (hazard ratio 1.83; 95% confidence interval [CI] 1.10–3.04; *P* = .020) but had no significant difference after multivariate adjustment (adjusted hazard ratio 1.65; 95% CI 0.91–2.97; *P* = .1.00) (Table 2).

**Table 2:** Primary and secondary outcomes stratified by self-identified race

	Black	Non-Black	*P* value
No. of patients	58	200	
VT recurrence	23 (39.7)	43 (21.5)	.009
Left ventricular assist device	4 (6.9)	7 (3.5)	.273
Heart transplant	4 (6.9)	4 (2.0)	.079
All-cause death	7 (12.1)	30 (15.1)	.718
Composite events	14 (24.1)	36 (18.2)	.413
Crude hazard ratio for VT recurrence	1.83 (1.10–3.04)	Ref	.020
Adjusted∗ hazard ratio for VT recurrence	1.65 (0.91–2.97)	Ref	.100
Crude hazard ratio for all-cause death	0.56 (0.25–1.34)	Ref	.200
Adjusted∗ hazard ratio for all-cause death	0.49 (0.21–1.17)	Ref	.107
Crude hazard ratio for composite event	1.28 (0.69–2.38)	Ref	.431
Adjusted∗ hazard ratio for composite event	0.76 (0.37–1.54)	Ref	.444

Cox proportional hazard models for ventricular tachycardia (VT) recurrence and composite events by self-identified race. Values are given as n (%) or hazard ratio (95% confidence interval) unless otherwise indicated.

∗ Results are adjusted for age, sex, history of hypertension, history of chronic kidney disease, presentation as VT storm, and total procedural time.

### All-cause death, LVAD, and heart transplant

**Figure 2:**
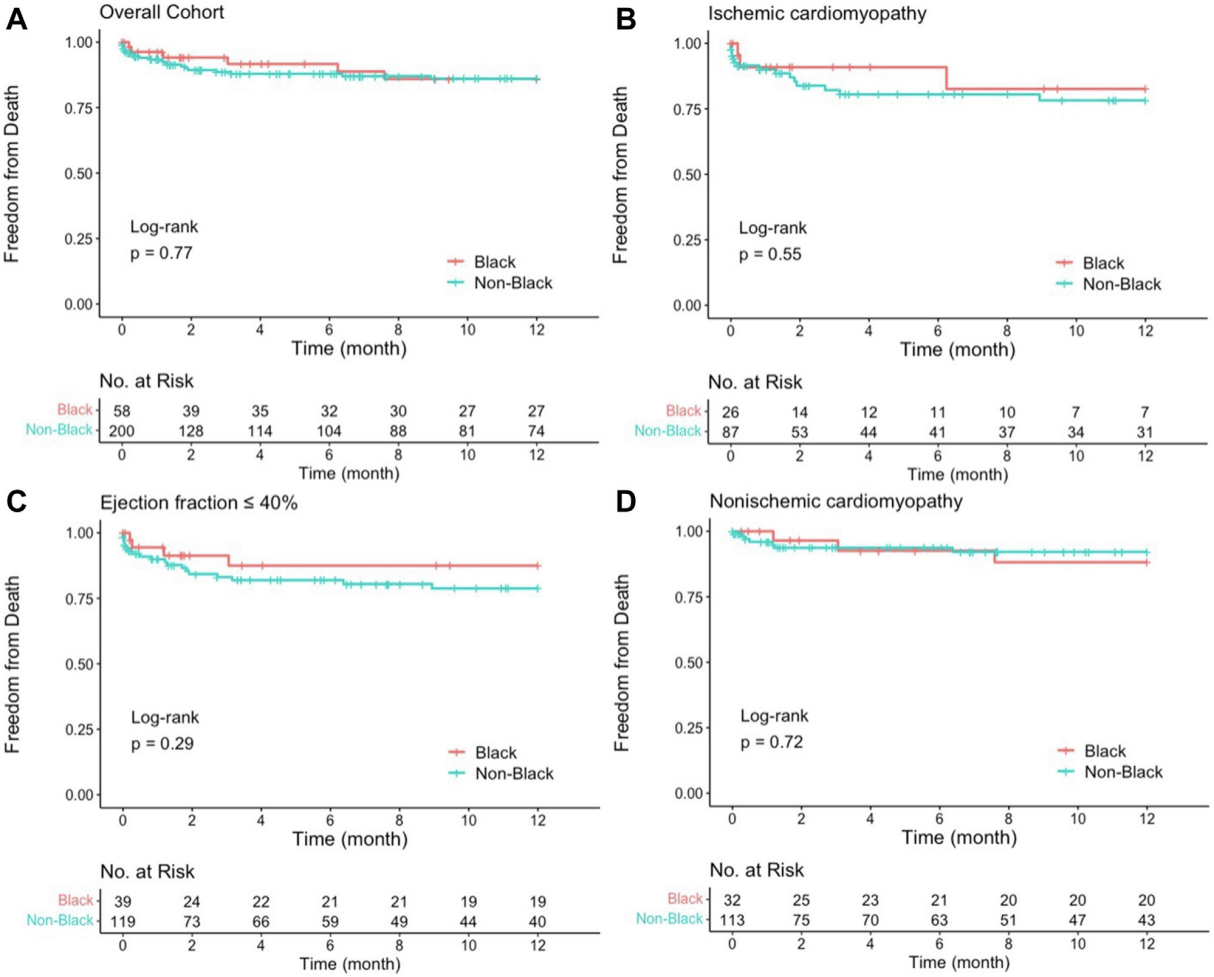
Freedom from all-cause mortality by self-identified race **(A)** and subgroup analysis of ejection fraction ≤40% **(B),** ischemic cardiomyopathy **(C),** and nonischemic cardiomyopathy **(D).**

**Figure 3:**
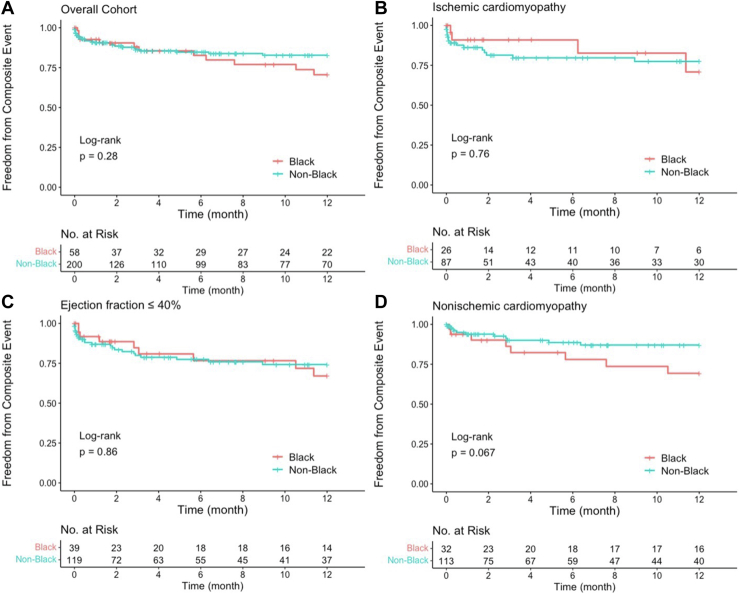
Composite event-free survival by self-identified race **(a)** and subgroup analysis of ejection fraction ≤40% **(B),** ischemic cardiomyopathy **(C),** and nonischemic cardiomyopathy **(D).**

Black patients had no difference in rates of all-cause mortality (12% vs 15%; *P* = .718) or rates of composite events (24% vs 18%; *P* = .413) compared to non-Black patients (Table 2). There was no statistically significant difference in crude or multivariate adjusted hazard ratios of all-cause death or composite events between races (Table 2). There was no significant difference in all-cause mortality between self-identified races in the year after ablation in the overall cohort and subgroup analysis (Figure 2). No differences were observed in the overall cohort or subgroups in the year after ablation between races for risk of composite events (Figure 3).

## Discussion

In this single-center prospective registry of consecutive patients undergoing scar-related VT catheter ablation at a tertiary academic medical center in south metropolitan Chicago, we found that patients who self-identified as Black were significantly more likely to experience VT recurrence after catheter ablation. However, after multivariate adjustment, no racial disparities in outcomes were observed. Additionally, the increased rate of VT recurrence was not at the expense of higher mortality or composite outcomes of death, transplant, or LVAD. This is one of the first analyses of VT ablation outcomes stratified by race and adds to the growing body of literature highlighting racial disparities in outcomes across the spectrum of cardiovascular disease.^8^, ^9^, ^10^, ^11^ The results of this study are similar to previous studies showing differences in outcomes by races from other complex arrhythmia ablations, particularly atrial fibrillation.^5^

There are a few potential explanations for the relationship seen in this study. First, Black patients may have been more likely to present or be referred late, as they more often presented with VT storm compared to non-Black patient (57% vs 40%; *P* = .028). However, the number of previous VT ablations and previous medication usage were not different between the races. Second, VT ablation in Black patients seemed to be more technically challenging with more extensive substrate, with trends toward longer total procedural times (300 vs 289 minutes), longer fluoroscopy time (25 vs 19 minutes), and longer ablation times (1672 vs 1423 seconds). There was no statistically significant difference when stratified by International Ventricular Tachycardia (I-VT) risk score (Table 1).^12^ However, 22% of Black patients were considered at high risk for VT recurrence by the I-VT score compared to 18% of non-Black patients. Similarly, 31% of Black patients were considered at high risk for mortality compared to 22% of non-Black patients. Additionally, the proportion of NICM, which traditionally portends worse outcomes, was similar between the races. Importantly, Black patients had a significantly higher prevalence of comorbidities, specifically hypertension (66% vs 40%) and CKD (55% vs 25%). This finding suggests that comorbid conditions such as hypertension and CKD may play a role in VT substrates as well as outcomes after VT ablation. This is especially true given that after adjustment for rates of hypertension, CKD, presentation of VT storm, and total procedural time, there was no statistically significant difference in VT recurrence. It is worth highlighting that there were no differences in other clinical outcomes such as all-cause mortality or rates of heart transplant or LVAD implantation. In fact, Black patients had a trend toward improved survival compared to non-Black patients (adjusted hazard ratio 0.49; 95% CI 0.21–1.17; *P* = .107).

When stratified by etiology of cardiomyopathy, Black patients were more likely to have VT recurrence in the ICM group only. We hypothesize that the higher rates of hypertension and CKD in Black patients were more likely to contribute to VT recurrence in the ICM group because both comorbidities are known factors that increase morbidity and mortality in patients with ICM.^13^^,^^14^ Additionally, comorbidities such as hypertension, CKD, and obesity have been shown to increase the risk of recurrence after other catheter ablations, such as pulmonary vein isolation for atrial fibrillation.^15^, ^16^, ^17^

In patients with NICM, no differences by race were observed in VT recurrence or all-cause death. However, there was a trend toward decreased freedom from composite events in Black patients vs non-Black patients (*P* = .067) (Figure 3). Additionally, there were trends toward higher rates of heart transplant (7% vs 2%) and LVAD implantation (7% vs 4%) between Black patients and other races within the overall cohort. These findings likely reflect the hypothesis that Black patients had more advanced cardiomyopathies at the time of catheter ablation.

Given that race in VT study populations is rarely reported, to the best of our knowledge this is the first cohort analysis to examine VT outcome differences by race. The University of Chicago Medical Center is distinctive in that is serves the local south metropolitan community with a high proportion of Black patients and whose primary insurer is Medicaid. Median household income of the top 5 zip codes served at the University of Chicago Medical Center is $37,948. Additionally, previous estimates indicate that >60% of health care encounters at the University of Chicago Medical Center are with Black patients and 64% are with patients whose primary insurer is Medicaid.^18^ Despite this, the percentage of Black patients in our cohort was 22%, which is significantly lower than across the University of Chicago Medical Center, with previous estimates indicating that >60% of health care encounters are with Black patients.^18^ The reason for this disproportionate referral pattern warrants further investigation. Overall, these data highlight the need for improved access to advanced VT therapies to reduce disparities in outcome, even with higher rates of comorbidities.

### Study limitations

First, our study was conducted from a single center with a unique patient demographic at a tertiary referral site, thus making the external generalizability limited. Larger multicenter series have not previously captured race. Second, patients were enrolled in the study at the time of catheter ablation but had variable and limited follow-up. Thirty-one patients (13%) were lost to follow-up, defined as <7 days of follow-up without an event. We attempted to mitigate loss to follow-up through regular phone calls and remote implantable device monitoring. Lastly, although we attempted to adjust for known covariates, there likely remain unknown confounders that may play a part in the differences observed in our study population. Multivariate adjustment does not necessarily separate the impact of medical and biological comorbidities from race and socioeconomic equity.

## Conclusion

In this diverse prospective registry of patients undergoing catheter ablation for scar-related VT, Black patients experienced higher rates of VT recurrence compared to non-Black patients. When adjusted for highly prevalent HTN, CKD, and VT storm, Black patients had comparable outcomes as non-Black patients.
